# Supplementary figures and images for: Choclo virus (CHOV) recovered from deep metatranscriptomics of archived frozen tissues in natural history biorepositories
Source: PLoS Negl Trop Dis. 2024 Jan 12;18(1):e0011672. doi: 10.1371/journal.pntd.0011672 (PMC10810438; doi:10.1371/journal.pntd.0011672)

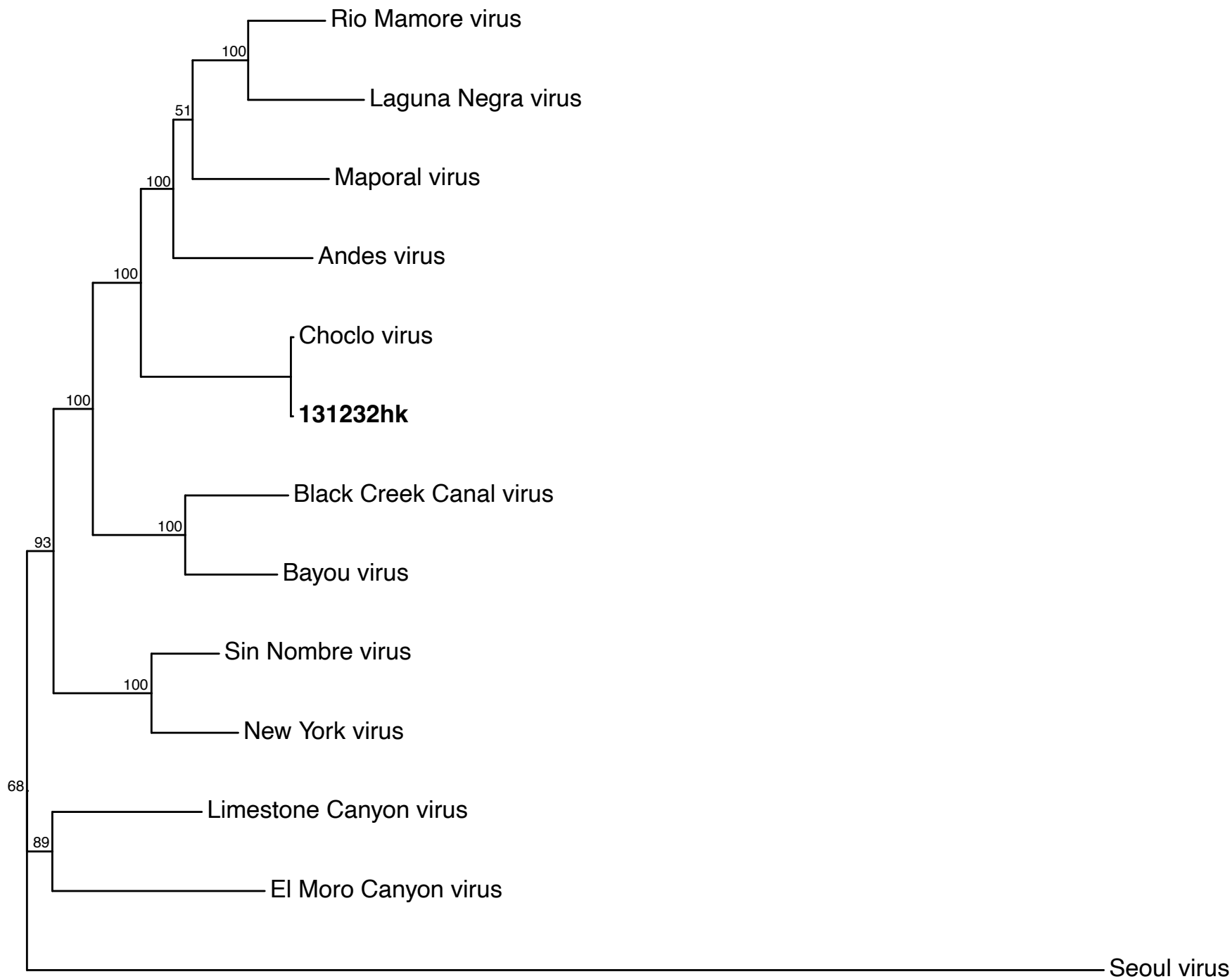

0.2

Supplement: S1 Fig — The phylogeny was built with the GTR+GAMMA model with 10,000 ultrafast bootstraps and 10,000 bootstraps for the SH-aLRT. LANV, BCCV, ELMCV, and NYV were limited to just the complete S and M segments. (PDF) [file pntd.0011672.s004.pdf]

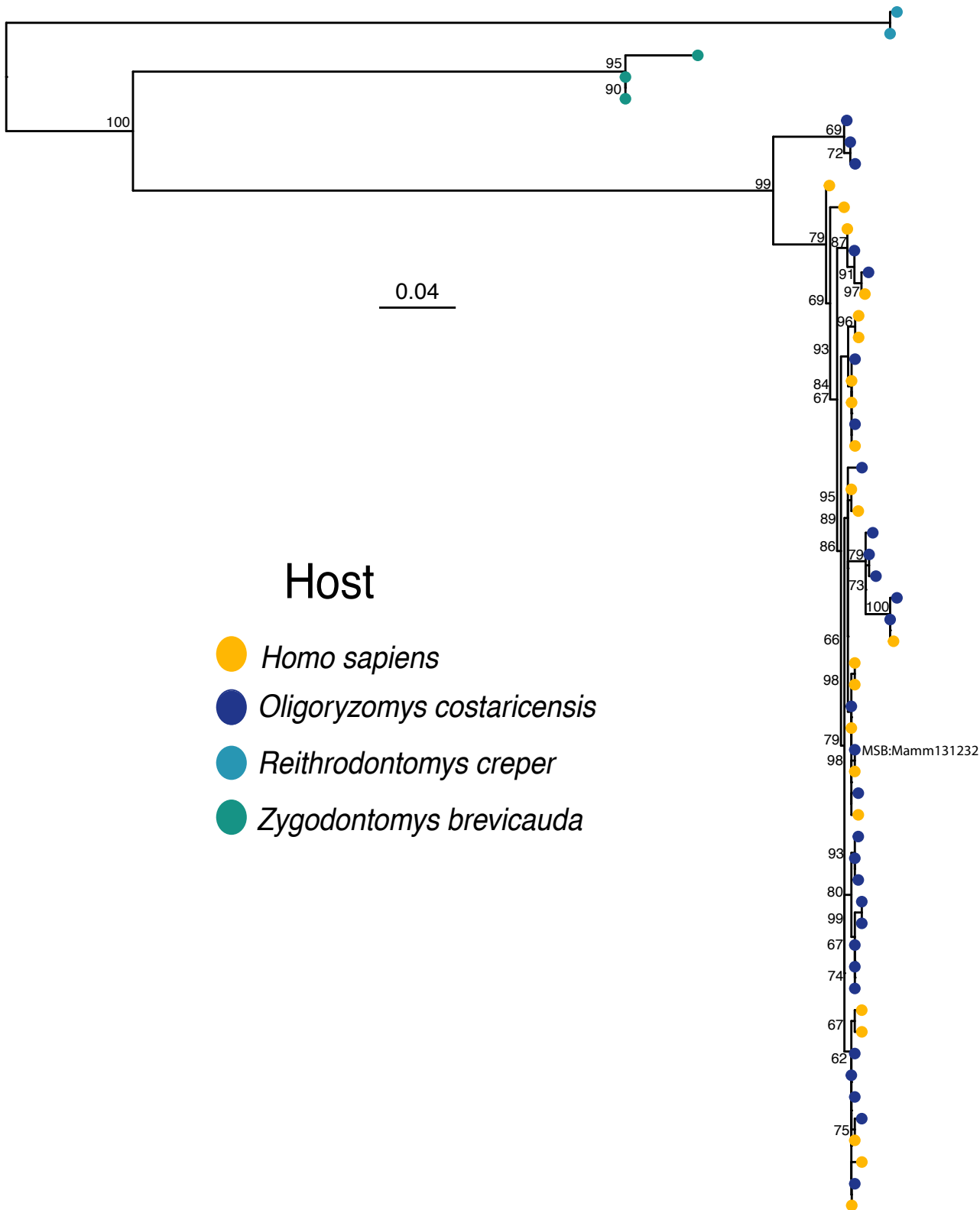

Supplement: S2 Fig — Sequences were obtained from sick human patients and three rodent hosts (Oligoryzomys costaricensis, Zygodontomys brevicauda, and Reithrodontomys creper). (PDF) [file pntd.0011672.s005.pdf]
