# Supplementary material for: Choclo virus (CHOV) recovered from deep metatranscriptomics of archived frozen tissues in natural history biorepositories
Source: PLoS Negl Trop Dis. 2024 Jan 12;18(1):e0011672. doi: 10.1371/journal.pntd.0011672 (PMC10810438; doi:10.1371/journal.pntd.0011672)
Supplement: Attachment — Submitted filename: Comments to technical review.docx [file pntd.0011672.s007.docx]

1) Please ensure that the funders and grant numbers match between the Financial Disclosure field and the Funding Information tab in your submission form. Note that the funders must be provided in the same order in both places as well. Currently, order of grants are different in both places.

NOTE: Please verify with the Funding Information Statement in the Online Submission Form.

**Thank you for bringing this to our attention. We have confirmed that information in the Funding statement and the online submission form are now the same.**

2) Thank you for stating, “All sequence data generated in this study were submitted to public archives. Reads were deposited in NCBI Short Read Archive (SRA) under BioProject PRJNA1015235. Host mitochondrial cytochrome b sequence was deposited under GenBank accession OR365535. The recovered Choclo virus assembly was deposited in NCBI GenBank under accessions OR365536, OR365537, OR365538.”

Please note that, though access restrictions are acceptable now, your entire data will need to be made freely accessible if your manuscript is accepted for publication. This policy applies to all data except where public deposition would breach compliance with the protocol approved by your research ethics board. If you are unable to adhere to our open data policy, please kindly revise your statement to explain your reasoning and we will seek the editor's input on an exemption. Please be assured that, once you have provided your new statement, the assessment of your exemption will not hold up the peer review process.

**We will adhere to the open data policy, and we have notified GenBank to release all sequence data.**

3) "Some material included in your submission may be copyrighted. According to PLOS’s copyright policy, authors who use figures or other material (e.g., graphics, clipart, maps) from another author or copyright holder must demonstrate or obtain permission to publish this material under the Creative Commons Attribution 4.0 International (CC BY 4.0) License used by PLOS journals. Please closely review the details of PLOS’s copyright requirements here: PLOS Licenses and Copyright. If you need to request permissions from a copyright holder, you may use PLOS's Copyright Content Permission form.

Please respond directly to this email and provide any known details concerning your material's license terms and permissions required for reuse, even if you have not yet obtained copyright permissions or are unsure of your material's copyright compatibility. Once you have responded and addressed all other outstanding technical requirements, you may resubmit your manuscript within Editorial Manager.

Potential Copyright Issues:

[Figure 3]: please (a) provide a direct link to the base layer of the map (i.e., the country or region border shape) and ensure this is also included in the figure legend; and (b) provide a link to the terms of use / license information for the base layer image or shapefile. We cannot publish proprietary or copyrighted maps (e.g. Google Maps, Mapquest) and the terms of use for your map base layer must be compatible with our CC BY 4.0 license.

Note: if you created the map in a software program like R or ArcGIS, please locate and indicate the source of the basemap shapefile onto which data has been plotted.

If your map was obtained from a copyrighted source please amend the figure so that the base map used is from an openly available source. Alternatively, please provide explicit written permission from the copyright holder granting you the right to publish the material under our CC BY 4.0 license.

If you are unsure whether you can use a map or not, please do reach out and we will be able to help you. The following websites are good examples of where you can source open access or public domain maps:
* U.S. Geological Survey (USGS) - All maps are in the public domain. (<http://www.usgs.gov>)
* PlaniGlobe - All maps are published under a Creative Commons license so please cite “PlaniGlobe, <http://www.planiglobe.com>, CC BY 2.0” in the image credit after the caption. (<http://www.planiglobe.com/?lang=enl>)
* Natural Earth - All maps are public domain. (<http://www.naturalearthdata.com/about/terms-of-use/>)

**Thank you, we have provided additional information in both the methods and the figure caption to indicate the source of the basemap shapefile and public data used to generate Figure 3. Please let me know if there are any additional issues.**
